# Integrating Biodiversity Infrastructure into Pathogen Discovery and Mitigation of Emerging Infectious Diseases

**DOI:** 10.1093/biosci/biaa064

**Published:** 2020-06-24

**Authors:** Joseph A Cook, Satoru Arai, Blas Armién, John Bates, Carlos A Carrion Bonilla, Maria Beatriz de Souza Cortez, Jonathan L Dunnum, Adam W Ferguson, Karl M Johnson, Faisal Ali Anwarali Khan, Deborah L Paul, DeeAnn M Reeder, Marcia A Revelez, Nancy B Simmons, Barbara M Thiers, Cody W Thompson, Nathan S Upham, Maarten P M Vanhove, Paul W Webala, Marcelo Weksler, Richard Yanagihara, Pamela S Soltis

**Affiliations:** Museum of Southwestern Biology and with the Biology Department, University of New Mexico, Albuquerque; Infectious Disease Surveillance Center, National Institute of Infectious Diseases, Tokyo, Japan; Departamento de Invetigación de Enfermedades Emergentes y Zoonóticas, Instituto Conmemorativo Gorgas de Estudios de la Salud, Panama City, Republic of Panama; Negaunee Integrative Research Center, The Field Museum of Natural History, Chicago, Illinois; Museum of Southwestern Biology and with the Biology Department, University of New Mexico, Albuquerque, and with the Museo de Mastozoologia QCAZ, Universidad Catolica del Ecuador, Quito, Ecuador; Florida Museum of Natural History, the UF Biodiversity Institute, and the Department of Biology, University of Florida, Gainesville; Museum of Southwestern Biology, University of New Mexico, Albuquerque; Gantz Family Collections Center, The Field Museum of Natural History, Chicago, Ilinois; Biology Department, University of New Mexico, Albuquerque; Faculty of Resource Science and Technology, Universiti Malaysia Sarawak, Jalan Datuk Mohammad Musa, Kota Samarahan, Sarawak, Malaysia; iDigBio and iDigInfo, Florida State University, Tallahassee; Department of Biology, Bucknell University, Lewisburg, Pennsylvania; Research Associate, Museum of Texas Tech University, Lubbock; Division of Vertebrate Zoology's Department of Mammalogy, American Museum of Natural History, New York, New York; William and Lynda Steere Herbarium, New York Botanical Garden, the Bronx, New York; Department of Ecology and Evolutionary Biology and with the Museum of Zoology, University of Michigan, Ann Arbor; School of Life Sciences, Arizona State University, Tempe; Centre for Environmental Sciences, Research Group Zoology: Biodiversity and Toxicology, Hasselt University, Diepenbeek, Belgium; Department of Forestry and Wildlife Management, Maasai Mara University, Narok, Kenya; Departamento de Vertebrados, Museu Nacional, Universidade Federal do Rio de Janeiro, Rio de Janeiro, Brazil; Pacific Center for Emerging Infectious Diseases Research, the John A. Burns School of Medicine, University of Hawaii, Manoa, Honolulu, Hawaii; Florida Museum of Natural History and with the UF Biodiversity Institute, University of Florida, Gainesville

The global human suffering, economic damage, and social disruption we are currently experiencing from the COVID-19 pandemic stem from inadequate preparedness and ineffective response to emerging pathogens. At its core, the COVID-19 pandemic is a consequence of our fundamental ignorance of our planet's natural ecosystems and the effects of our encroachment on them. Our reactive approaches to the emergence of zoonotic pathogens, which are responsible for approximately 75% of all new emerging infectious disease outbreaks, are too often based on limited knowledge of the origin, pathogenicity, and basic biology of the wild host and pathogen coupled with poor communication among relevant stakeholders. Others have pointed to this ignorance of viral diversity and offered solutions (Andersen et al. 2020), but a broad, fully integrative discussion of how to leverage existing infrastructure and to build new resources has been missing. In the present article, we call for the development of alternative tactics that are aimed at proactively meeting the daunting challenges to humanity posed by emerging zoonotic pathogens.

The potential role of natural history specimens in pathogen discovery and mitigation is recognized in the museum world (DiEuliis et al. 2016, Dunnum et al. 2017) and by at least some disease ecologists (e.g., Mills and Childs 1998). However, relatively few in the One Health community (e.g., Kelly et al. 2020) embrace the value of leveraging existing biodiversity infrastructure (i.e., natural history collections, biorepositories, and their associated expertise and informatics resources) to more fully understand zoonotic pathogen emergence and reemergence. This concept is not new; in the early 1900 s, the American Museum of Natural History created the Department of Public Health (Brown 2014). Although a lack of funding put an early end to the initiative, the Department of Public Health made extensive progress, from clever exhibitions for the public to assembling a living collection of bacterial cultures (Brown 2014). Renewed efforts to align pathobiology with biodiversity discovery initiatives are critical. Moreover, linking both biodiversity infrastructure and building capacity closer to zoonotic pathogen surveys in biodiverse countries would substantially improve proactive responses to pandemics before they once again wreak havoc across the globe.

## Biodiversity science as a tool in biomedical research and response

Earth's biodiversity is connected through a single evolutionary tree of life, and pathogens (whether viruses, bacteria, or eukaryotes) and their hosts represent millions of years of evolutionary interactions. Medical researchers have long used this knowledge to advance our understanding of how certain microbes cause disease in humans. For example, because fundamental aspects of malaria parasitism are extremely difficult to study in humans, New World monkeys—­particularly, owl monkeys in the genus Aotus—have been important models for studying strains of malaria to develop vaccines, some of which are now in clinical trials. Taxonomic research based on museum specimens (Hershkovitz 1983) demonstrated that geographically separated species of owl monkeys have varying tolerance to the parasite and that the failure to recognize these taxonomic differences can hamper research. We have only begun to understand how widespread and diverse coronaviruses are in nature, and important gaps in regional and phylogenetic coverage persist (Anthony et al. 2017). Understanding their functional interactions with host cells and developing the most effective strategies to combat pathogenic coronaviruses will require documenting genetic relationships of the virus and among the wild hosts (Andersen et al. 2020). Archiving these associations in accessible and curated specimen databases is crucial now and into the future (e.g., www.globalbioticinteractions.org). Building on a solid foundation of knowledge of evolutionary and ecological relationships of hosts and pathogens enables scientists to possibly predict the emergence of future zoonotic diseases and to respond to novel outbreaks more rapidly and efficiently (Brooks et al. 2019).

## The need to strengthen biodiversity infrastructure and increase discovery

The detection and description of novel pathogens usually requires large numbers of host samples because of low prevalence (Plowright et al. 2019). The world's natural history collections contain more than 3 billion specimens. Although the vast majority of these specimens may not be suitable for pathogen discovery, specimens provide a powerful roadmap to the spatial and temporal distribution of global biodiversity. A growing trend in many museums (e.g., www.idigbio.org/content/dna-banks-and-genetic-resources-repositories-united-states, www.ggbn.org) is the establishment of cryopreserved biorepositories, including vertebrate samples that often preserve associated parasites. These collections represent multiple, diverse host samples archived broadly across space and time that could readily be probed for pathogens. More commonly, however, novel pathogen discovery involves field surveys of wild hosts. Unless a particular pathogen is targeted, survey strategies that focus on taxonomically diverse species across spatially broad distributions provide the best opportunities for detection. Typically, field surveys of terrestrial vertebrates are noninvasive (using swabs or fecal samples) and do not produce archived specimens, so they rarely contribute to the shared biodiversity infrastructure of the world's scientific community. By instead linking these field surveys to permanent natural history collections, future pathogen discovery would be connected more broadly to other avenues of biodiversity research and naturally promote integration and synergy across scientific disciplines.

An additional benefit from closer ties between pathobiology and natural history collections involves the voucher concept. Biodiversity studies, when possible, should be backed by a permanent sample or voucher, which would facilitate replication and validation, extension, and integration across disciplines (Cook et al. 2016, Lendemer et al. 2020). To date, few of the published nonhuman betacoronavirus sequences are tied to a permanent sample that would allow implementation of these central tenets of the scientific method (but see Joffrin et al. 2020). A change in practice, through improved communication between biodiversity and biomedical scientists, would both enhance the quality of any data collected from the pathogen and add value by enabling future analyses of the genotype, phenotype, and interactions of the same pathogen source.

In addition to serving as permanent archives and providing samples for research, natural history collections and their associated biorepositories provide expertise in taxonomy, identification, phylogenetics, niche modeling, evolutionary dynamics, and other knowledge critical to pathogen monitoring, mitigation, and control. In the past few decades, museums have become hubs of biodiversity informatics, serving as the critical nexus between biological samples and sample-derived data (e.g., genomics, geographic information, isotope chemistry, CT scans). The current pandemic reminds us that natural history specimens are important but underappreciated reservoirs for studying the hosts and distributions of animal and human pathogens (see Harmon et al. 2019) and that the data connected to these specimens increase our understanding not only of the host organism but of the pathogens as well. Enhanced support of both physical and cyberinfrastructure for biodiversity collections would yield an information system to enable prediction and mitigation of future outbreaks and pandemics.

The most biodiverse places on the planet occur in developing countries, so there is a huge need to develop local and regional capacity and scientific expertise in biodiversity research and collections. International scientific partnerships aiming to increase research transfer and building local capacity will help to match resources and technology available in developed countries. Therefore, it will facilitate early detection and mitigation in front of an outbreak. Given the tremendous need to understand how human-mediated loss of biodiversity and transformation of natural ecosystems will affect human health, building human capacity and strengthening ties between research and clinical infrastructure in developing countries is imperative.

## Informatics as a tool for disseminating knowledge

Natural history institutions have produced extensive digital data and continue to digitize information from their physical collections. Online scientific databases (e.g., iDigBio, GBIF, VertNet, Arctos, Atlas of Living Australia, SpeciesLink) serve as portals to natural history archives, offering researchers around the world access to data and metadata (including linked genetic, environmental, and other information) associated with vouchered specimens. Furthermore, the development of this cyber-enabled information system is crucial for understanding our natural world and the relationships between biodiversity and human health. Connecting natural history archives and pathobiology is not only necessary but easier to achieve today than ever before. For example, free, online access to global specimen data provides efficient opportunities for loans of physical specimens from museums to biomedical laboratories for analysis of pathogens. Surprisingly, the robust cyberinfrastructure supporting living stock collections—which make viral, bacterial, and other pathogen lines and samples available to the biomedical research community—is not connected to that of natural history collections. These communities are only vaguely aware of each other's resources, despite obvious benefits for both basic and clinical research. However, a clear, long-term pathway must be implemented so that pathobiologists are fully aware of the varied resources available in natural history collections and can use and contribute to these resources.

## A new vision for predicting and responding to pandemics

The twenty-first century has already seen multiple major new disease ­outbreaks—from SARS and MERS to Ebola and Zika—culminating in the current COVID-19 pandemic. What have we learned from these events, and how do we harness that knowledge for prediction and response? Ongoing encroachment by humans into natural ecosystems will continue to promote contact with potential pathogens. Absent global cooperation to restrict further habitat degradation and eliminate illegal wildlife trade, we need new approaches to gather, share, and interpret data and knowledge for deployment in preventing, predicting, and responding to future pandemics. We suggest five key elements as a framework for research and future resilience.

Best practices must be developed for sample preparation. Biodiversity scientists, collections managers, disease ecologists, and microbiologists must converge on common guidelines for sampling, preserving, and archiving samples of both pathogens and hosts to ensure reproducible science and future access to samples studied in a particular context. Vouchering of host materials and pathogen preparations will require expanded capabilities in natural history collections and biorepositories, and cooperation among communities will be needed to ensure space and adequate curation of materials.

Metadata requirements must be developed to accompany the physical specimens and samples collected, analyzed, and archived. The essential elements will be the application of universally unique identifiers for all specimens and their derivative products—including tissues, pathogen preparations, genetic sequences, and beyond. Again, communication among communities, including museum personnel, biomedical researchers, and personnel at global genetic databases, will be crucial for identifying and adopting metadata that will enhance the value of biological materials.

Infrastructure, both physical and cyber, is required to support both current and future biological materials, whether in natural history collections, living stock collections, or other biorepositories. Because our knowledge of potential emerging pathogens is so limited and because the pathogens themselves evolve and diversify, we recommend expanded collection of field samples of organisms that are likely reservoirs of zoonotic diseases and other associated possible hosts. The preparation of these materials following the first element above will require expanded capacity and implementation of new curation methods in many institutions. Likewise, further investment in cyberinfrastructure to link together all known data and knowledge related to specimens, genetics, environment, literature, and more would enhance responses to future disease outbreaks.

Perhaps the most important but most difficult element is the adoption and implementation of practices that change how a community conducts its science. We endorse open science concepts and practice and advocate increased communication and the development of new channels of dialogue and collaboration. This is particularly relevant within the integrative approaches to health that have increasingly become adopted, because they draw from multiple contributing sciences and sectors (Lerner and Berg 2017).

The implementation of these elements requires strong leadership and financial support from a range of federal agencies, international partners, and private foundations worldwide to provide infrastructure and enable development of proactive approaches to future pandemics. Because the assets and returns are substantial for science, policy, and human well-being alike, we recommend that both research funders and the providers of official development aid engage in this effort. Such investment, even on the scale needed to accomplish the goals outlined in the present article, would be inconsequential compared to the loss of life and the economic catastrophe brought by COVID-19. Many of the pieces of our emerging vision are already in place, but a more resilient and integrated initiative that leverages and builds existing biodiversity infrastructure is critically needed.

## Author Biographical

Joseph A. Cook is affiliated with the Museum of Southwestern Biology and with the Biology Department at the University of New Mexico, in Albuquerque. Satoru Arai is affiliated with the Infectious Disease Surveillance Center at the National Institute of Infectious Diseases, in Tokyo, Japan. Blas Armién is affiliated with the Departamento de Invetigación de Enfermedades Emergentes y Zoonóticas at the Instituto Conmemorativo Gorgas de Estudios de la Salud, in Panama City, Republic of Panama. John Bates is affiliated with the Negaunee Integrative Research Center at The Field Museum of Natural History, in Chicago, Illinois. Carlos A. Carrion Bonilla is affiliated with the Museum of Southwestern Biology and with the Biology Department at the University of New Mexico, in Albuquerque, and with the Museo de Mastozoologia QCAZ, at the Universidad Catolica del Ecuador, in Quito, Ecuador. Maria Beatriz de Souza Cortez is affiliated with the Florida Museum of Natural History, the UF Biodiversity Institute, and the Department of Biology at the University of Florida, in Gainesville. Jonathan L. Dunnum is affiliated with the Museum of Southwestern Biology at the University of New Mexico, in Albuquerque. Adam W. Ferguson is affiliated with the Gantz Family Collections Center, at The Field Museum of Natural History, in Chicago, Ilinois. Karl M. Johnson is affiliated with the Biology Department at the University of New Mexico, in Albuquerque. Faisal Ali Anwarali Khan is affiliated with the Faculty of Resource Science and Technology at the Universiti Malaysia Sarawak, Jalan Datuk Mohammad Musa, Kota Samarahan, in Sarawak, Malaysia. Deborah L. Paul is affiliated with iDigBio and iDigInfo, at Florida State University, in Tallahassee. DeeAnn M. Reeder is affiliated with the Department of Biology at Bucknell University, in Lewisburg, Pennsylvania. Marcia A. Revelez is a Research Associate at the Museum of Texas Tech University, in Lubbock. Nancy B. Simmons is affiliated with the Division of Vertebrate Zoology's Department of Mammalogy at the American Museum of Natural History, in New York, New York. Barbara M. Thiers is affiliated with the William and Lynda Steere Herbarium, at the New York Botanical Garden, in the Bronx, New York. Cody W. Thompson is affiliated with the Department of Ecology and Evolutionary Biology and with the Museum of Zoology at the University of Michigan, in Ann Arbor. Nathan S. Upham is affiliated with the School of Life Sciences at Arizona State University, in Tempe. Maarten P. M. Vanhove is affiliated with the Centre for Environmental Sciences in the Research Group Zoology: Biodiversity and Toxicology, at Hasselt University, in Diepenbeek, Belgium. Paul W. Webala is affiliated with the Department of Forestry and Wildlife Management at Maasai Mara University, in Narok, Kenya. Marcelo Weksler is affiliated with the Departamento de Vertebrados, Museu Nacional, at the Universidade Federal do Rio de Janeiro, in Rio de Janeiro, Brazil. Richard Yanagihara is affiliated with the Pacific Center for Emerging Infectious Diseases Research, in the John A. Burns School of Medicine, at the University of Hawaii at Manoa, in Honolulu, Hawaii. Pamela S. Soltis (psoltis@flmnh.ufl.edu) is affiliated with the Florida Museum of Natural History and with the UF Biodiversity Institute, at the University of Florida, in Gainesville.
